# Observations on Piperine, with the Formula for Its Preparation, &c.

**Published:** 1828-08

**Authors:** George W. Carpenter

**Affiliations:** Philadelphia.


### PIPERINE.

### *Observations on Piperine, with the Formula for its Preparation, &c.*

By George W. Carpenter, of Philadelphia.*

The object of the present communication is to describe a new principle, recently discovered in black pepper, which has been denominated Piperine, and which has been proved, by careful experiment, to be an active remedy in intermittent fevers, and has been employed with much advantage also in typhous fever and periodical headache, and, from the testimonials given in its support, seems to bid fair to become an important addition to the materia medica. It may be employed in doses of from one to four grains; it has been given in several cases of intermittent fevers in doses of one grain, and was attended with as much success as the quinine. It is found to be a valuable adjunct to that substance, equal parts of each acting with more energy and success than the whole quantity of quinine.

Black pepper, in its crude state, has long been known as a valuable medicine, and is stated to be an excellent adjunct to bark in intermittents; and Mr. Rennie† observes, that Mr. Brande must certainly be mistaken when he says it acts only as a warm condiment, agreeable to the stomach.

It is mentioned in Dr. Coxe's valuable Dispensatory, under the article Piper, that Dr. Frank, physician to her

* Condensed from the American Journal of Medical Sciences.

† See his Supplement to the Pharmacopœias of London, Edinburgh, Dublin, and Paris.

Majesty Maria Louisa, recommends the black pepper in different species of intermittent fevers. This had previously been used in the East with success, after every known means had been ineffectually tried. The dose is five to ten grains twice a day, and Dr. Ghigini reports ten cases cured with it. Dr. Frank mentions seventy patients who came under his notice between April and June, of whom fifty-two had tertian, ten quotidian, and eight the quartan fever. Fifty-four were completely cured within about a week, without any subsequent relapse. He dips the seeds of the black pepper in a mucilage of gum Arabic, and subsequently in powdered colomba, to disguise it, and gives from five to eight pills twice a day. None of his patients required more than from seventy to eighty pills for a complete cure. Dr. Frank recommends the profession to try the extract of black pepper in intermittent fevers. This preparation was tried on nine individuals affected with intermittent fevers of various types, in doses of four, eight, ten, or twelve grains, dissolved in water in some cases, and given in the form of pills in others, by Dr. Clock, of Trent; and the effects surpassed his warmest expectations.

From these experiments it is concluded, that the extract of pepper is not only one of the best succedaneums for the bark, but that it is even preferable to it on several accounts: 1st. It never produces disturbance in the stomach or bowels; 2d, it never failed in producing a cure; 3d, those who were cured did not in any one instance experience a relapse; 4th, it produces a regular alvine discharge, as well as the excretion of urine and sweat; 5th, none of those who were cured experienced that sensation of languor, so common to a state of convalescence.*

In the Bulletin des Sciences Medicales for April 1826, there is an account of three cases treated with piperine, by Dr. S. Gordoni, physician to the hospital at Leghorn. The first case was one of double tertian intermittent fever, which for a long time resisted the use of quinine, and, although this remedy eventually arrested the paroxysms, whenever it was discontinued the disease returned: the piperine was then resorted to, and the fever ceased on the first day, and did not return. The second and third were also cases of intermittent fever: they were promptly cured by the piperine. From these and many other cases, Dr. Gordoni infers that the piperine will cure fevers that resist the sulphate of quinine, and that it will prevent a relapse better than this latter remedy.

* Giornale de Chirurgia Practica.

Dr. Meli has also employed the piperine with success, and considers it a more certain remedy in intermittent fever than the sulphate of quinine.*

Dr. J. S. Rose, who was the first to use the piperine in this city, informs me that he has employed the piperine which I prepared in twenty cases of intermittent and remittent fevers, and that he is decidedly of opinion that it will be found to be a more certain and efficient remedy than any preparation of bark heretofore used.

"I have also used it (he adds,) in two cases of low nervous fever, or typhus. I was induced to employ it in these cases, from observing that in intermittent it did not prevent all the stages of the paroxysm. At the time the patient expected his chill, he found a gentle diaphoresis, which continued to increase for two, three, and in some cases for four hours: on the next day, however, of the expected return, there was nothing like diaphoresis or fever. The patient passed this period without the least inconvenience, and remained exempt from a relapse, which is not always the case after the use of quinine. These facts led me to believe that in typhus, when we wish a stimulating diaphoretic, no remedy is preferable for that purpose to the piperine, not even volatile alkali. In this form of febrile action, when the animal powers are about to yield to the influence of disease, and the patient fall a victim to the timidity of the practitioner, I have boldly withheld all other remedies, and administered the piperine in doses of two grains every two hours, until eight grains had been taken: the low muttering delirium now began to subside, the skin became moist, and the patient, sensible of his improvement, expressed himself better. On the following day the same doses were administered, and repeated for three, four, or five days, when I found no fever, the strength increased, and the patient with an inclination for food, and convalescent."

The piperine has been also used by Dr. J. C. Rousseau, of this city, in a few cases, in all of which it has been successful. He has favored me with the following cases, which are interesting, as it shews the necessity of caution in prescribing the remedy in large doses.

"A young girl, about twelve years of age, having had a return of an intermittent fever that had been stopped by the sulphate of quinine, was directed to take one grain of the piperine, made into a pill with conserve of roses. She was a short time after seized with a vomiting, which was repeated to the number of seven times in the space of two hours. It then began to promote alvine evacuations to the extent of twelve or fifteen times. The fever did not return, and she was directed to continue one grain of the me-

* Ainslie's Materia Indica, vol. ii. p. 622.

dicine night and morning. It invariably produced alvine discharges in an unusual quantity.

"In another case, a subject of about forty, it produced a radical cure, in the dose of three grains, in twenty-four hours, continued for several days after. And it is so much the more remarkable, as this patient had taken the sulphate of quinine for some days, in the quantity of thirty grains every twenty-four hours, as he informed me; remarking at the same time, that, during the use of it, he was under a most violent and painful state of excitement."

I have just received the following case from my friend, Dr. J. R. Black, of Philadelphia: it affords additional testimony of the powers of the piperine in the cure of intermittent fever.

"Mr. S., aged about forty years, during the first part of last month applied with a severe quotidian fever, attended with rejections from the stomach, and with violent pain and great determination of blood to the head during the hot stage, with cold feet and slight delirium.

"The case was treated with the lancet, emetics, and purges, which, on the third day, changed its type to the tertian. On the day of intermission, sulphate of quinine was administered, which was often rejected, while it always increased the patient's nausea and headache. Piperine (prepared by Mr. Carpenter,) was substituted in doses of one grain every hour, to the number of ten a day. The paroxysms immediately ceased, and the patient in a few days was discharged radically cured."

Many other cases might be quoted, in which this medicine has been employed with equally happy results, but enough has been advanced to satisfy the most sceptical of its active properties.

Alcohol and sulphuric æther are the best menstrua for the active properties of the pepper, which very soon imparts its acrimony to these fluids. Mr. Brande gives alcohol and water. I am surprised he should have omitted æther, since it is the most powerful solvent; and particularly that he should quote water, as it requires 550 pints to extract the sapidity of one pound of pepper. Water, indeed, appears to rank lowest as a solvent of the active part of the pepper, while it is the best solvent of the colouring matter; for, after the pepper has been exhausted of its sapidity by æther and alcohol, water will make a deep-coloured solution, which, on evaporation, will produce a dark extract, possessing little of the pungency of the pepper.

The piperine employed in the above cases I prepared according to the following formula:

Digest one pound of coarsely powdered black pepper in one gallon of alcohol for ten days; distil off one-half of the alcohol in a water bath; add by degrees diluted muriatic acid, to hold in solution the piperine; then add water sufficient to precipitate the resin and separate the oil, a muriate of piperine remaining in solution; concentrate this solution by evaporation, and add pure potass to decompose it, and neutralise the acid; when the piperine, in consequence of the diluted state of the alcohol, and the absence of the muriatic acid, will be deposited in yellowish transparent crystals. The crystals may be obtained perfectly colourless, by carefully separating the oil and resin; but, as there is no disadvantage in the colour, the additional trouble and expense would not be compensated. The piperine in a colourless state is insipid and inodorous, but, united with as much resin as enters into its crystallization, its taste is extremely hot, possessing in an intense degree all the pungency of the pepper, with a considerable portion of its odour, and I think is more active than the former. It was in this form exhibited in the treatment of the cases above described.

The crystals were perfectly transparent, tetrahedral prisms, with oblique summits of a straw colour, and as large as the ordinary crystals of sulphate of magnesia.

*Extract of Black Pepper.*—Digest eight ounces of black pepper, coarsely ground, in four pints of diluted alcohol for four days, occasionally submitting it to a temperature near ebullition in a water bath; filter and evaporate to the consistence of an extract.

This is found also to be an active remedy in intermittents, in doses of two or three grains. In a soft state it has proved very convenient to give consistency to piperine and quinine for the form of pills, while at the same time it increases their activity, particularly that of the latter. It is certainly preferable to conserve of roses or gum Arabic, as they enlarge the pill without increasing its effect.

The extract of pepper, in every formula I have seen, has been directed to be prepared with water. This forms a much less active preparation, and possesses several inconveniences, to which the former is not subject.

I have used both the white and black peppers in the above preparation, and although it is stated by most authors that the white is milder than the black, I have found it to yield more piperine, and an extract of much more acrimony and activity, and to contain much less colouring matter.

The constituent principles of pepper are piperine, oil, resin, fecula, and colouring matter.

The oil of black pepper is a most powerful article, and has not as yet been employed in medicine: it may prove a valuable stimulant.

